# Histoblast: A Novel Cavitative High-Intensity Focused Ultrasound (HIFU)-Modified Technique for Benign Prostatic Hyperplasia

**DOI:** 10.7759/cureus.98989

**Published:** 2025-12-11

**Authors:** Carlos M Garcia-Gutierrez, Carlos A Garcia-Becerra, Natalia Garcia-Becerra, Habid Becerra-Herrejon, Sunao Shoji, Narendra T Sanghvi

**Affiliations:** 1 Department of Urology, UroVallarta Urological Center, Puerto Vallarta, MEX; 2 Department of Research and Development, UroVallarta Urological Center, Puerto Vallarta, MEX; 3 Department of Urology, Tokai University School of Medicine, Kanagawa, JPN; 4 Department of Research and Development, Sonablate Corporation, Indianapolis, USA

**Keywords:** ablation technique, benign prostatic hyperplasia, high intensity focused ultrasound ablation, prostatic adenoma, ultrasonic surgical procedure

## Abstract

Benign prostatic hyperplasia (BPH) is a prevalent condition, often requiring intervention due to its impact on quality of life. High-intensity focused ultrasound (HIFU) has demonstrated efficacy in prostate cancer treatment; however, its application in BPH remains limited by prolonged treatment times and delayed symptom relief. This report introduces a novel HIFU-based technique (Histoblast) using the Sonablate 500® (SB-500) system to improve treatment efficiency and clinical outcomes in BPH. Key modifications to the conventional HIFU approach include: (1) a checkerboard treatment pattern to reduce treatment time (TT) and tissue edema; (2) prostate compression via increased degassed water volume to minimize heat dissipation and enhance acoustic efficiency; and (3) optimized energy delivery within the upper third of the device’s approved range. Treatment progress is monitored using standard safety parameters, including Tissue Change Monitoring and Reflectivity Index measurements. The procedure is performed under spinal anesthesia, with postoperative catheterization lasting approximately four days. This technique reduces the number of treatment shots by 40-50%, achieving a significantly shorter TT. Controlled acoustic cavitation is observed in up to 70-75% of treated zones, indicating effective tissue ablation. Preservation of the urethra and bladder neck is prioritized to facilitate early catheter removal. This technique represents a promising refinement of HIFU for BPH management and warrants further clinical evaluation.

## Introduction

Benign prostatic hyperplasia (BPH) affects an estimated 210 million men worldwide, with a prevalence ranging from 50% to 80% in individuals aged 50 to 80 years [1]. Given its high prevalence and significant impact on quality of life, there is ongoing innovation in the development of minimally invasive surgical therapies (MIST) aimed at improving treatment outcomes and reducing morbidity.

High-intensity focused ultrasound (HIFU) is an ablative therapy first introduced in the 1940s [2]. While HIFU has demonstrated favorable clinical outcomes in the treatment of prostate cancer [3], its application in BPH has shown more limited success. The main drawbacks cited in the literature include prolonged treatment time (TT), averaging approximately 10 cc of tissue ablation per hour [4], and delayed clinical improvement, primarily due to the gradual post-treatment elimination of necrotic tissue [5]. These factors contribute to extended catheterization durations, often lasting between 2 to 3 weeks, thereby limiting broader clinical adoption.

During the 1990s, several clinical trials [6,7] evaluated the use of HIFU for the treatment of BPH. While these studies demonstrated HIFU’s ability to ablate prostatic tissue, they also revealed several limitations, including prolonged TT, extended catheterization duration, and slow clinical improvement [8]. Some researchers hypothesized that this delayed clinical response was due to the slow elimination of necrotic tissue typically associated with thermal ablative techniques [8]. In studies of whole-gland HIFU treatment for prostate cancer, one of the most commonly reported adverse effects was bladder outlet obstruction, occurring in approximately 35.2% of cases [9,10]. This side effect is significantly less frequent in partial or focal HIFU therapies, reported in 15.8% [9] and 0-17% [11] of cases, respectively. The obstruction has been linked to edema caused by the high energy delivered during whole-gland treatments [10].

The aim of this study is to present a novel HIFU technique designed to optimize energy delivery and improve clinical outcomes in BPH, addressing key limitations of conventional HIFU treatment, such as prolonged TT, extended catheterization, and delayed recovery.

This technique was prospectively registered with the United States patent office (Patent no.: US 11,524,183 B1) and presented at major meetings as an abstract [12].

## Technical report

The procedure was performed using the Sonablate 500® (SB-500) device (Sonablate Corp., Indianapolis, USA), composed of two sections: the B-mode ultrasound system and the HIFU power generator, and a cooling system involving a degassed water flow.

In contrast to the conventional treatment described previously in the literature [4], this technique incorporates three key modifications: (1) a checkerboard treatment plan aimed at reducing TT and tissue edema; (2) prostate compression achieved by using a greater volume of degassed water than standard treatment, intended to minimize heat dissipation and lower the acoustic cavitation threshold; and (3) optimized energy delivery by consistently applying a configuration within the upper third of the energy range permitted by the device’s approved configuration for standard therapies.

The latter two modifications synergistically enhance acoustic pressure at the focal point, promoting more frequent acoustic cavitation, which may increase the efficiency of tissue ablation.

Procedure description

All patients follow a standardized preoperative protocol, including 12 hours of fasting and bowel preparation with laxatives. In the operating room, patients are positioned in lithotomy, fitted with a transurethral catheter and pneumatic compression stockings, and placed under spinal anesthesia. The transurethral catheter is inserted, leaving 200 mL of urine in the bladder (if the bladder is empty, saline is instilled).

**Figure 1:**
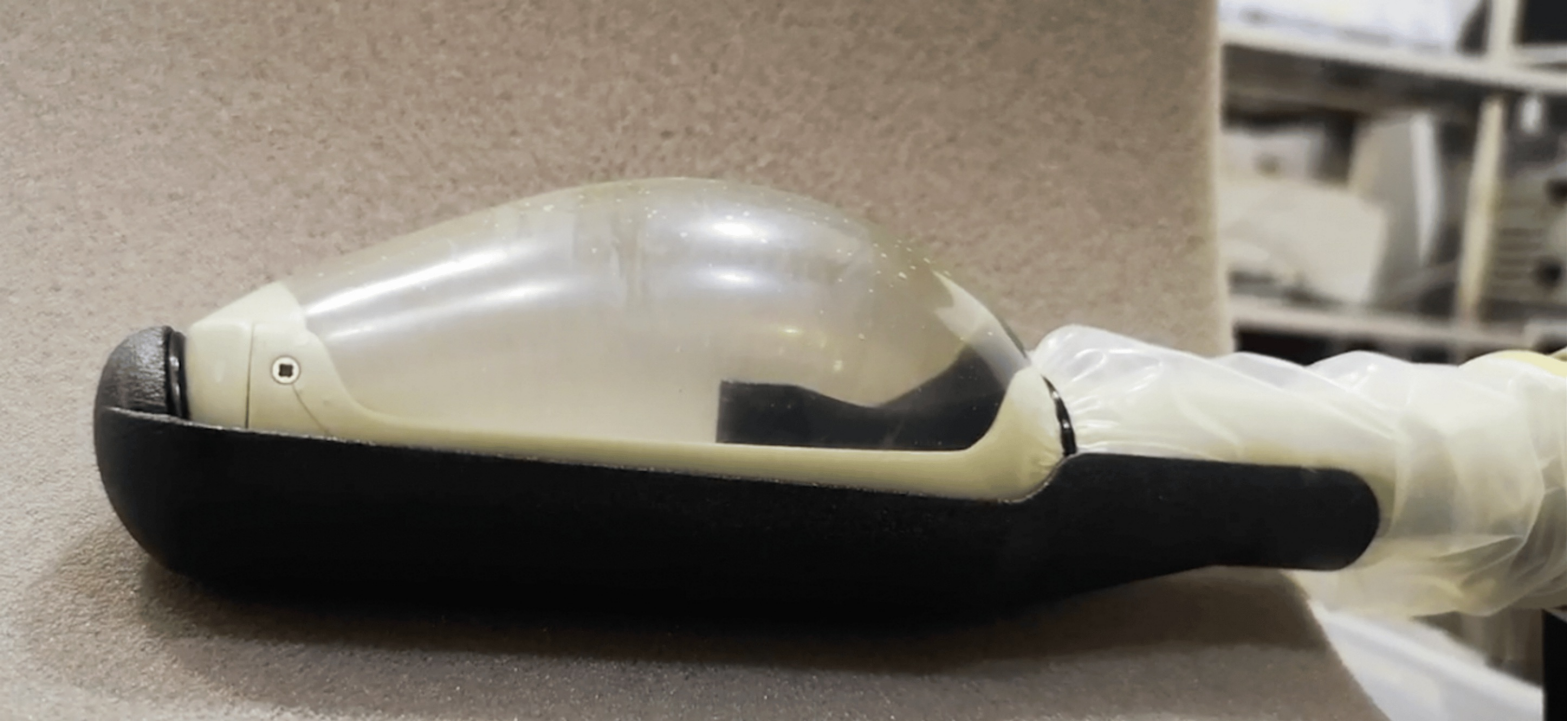
Representative image of the transducer filled with degassed water, used to produce prostate compression with the authors’ high-intensity focused ultrasound (HIFU) device. Image provided by the authors.

**Figure 2:**
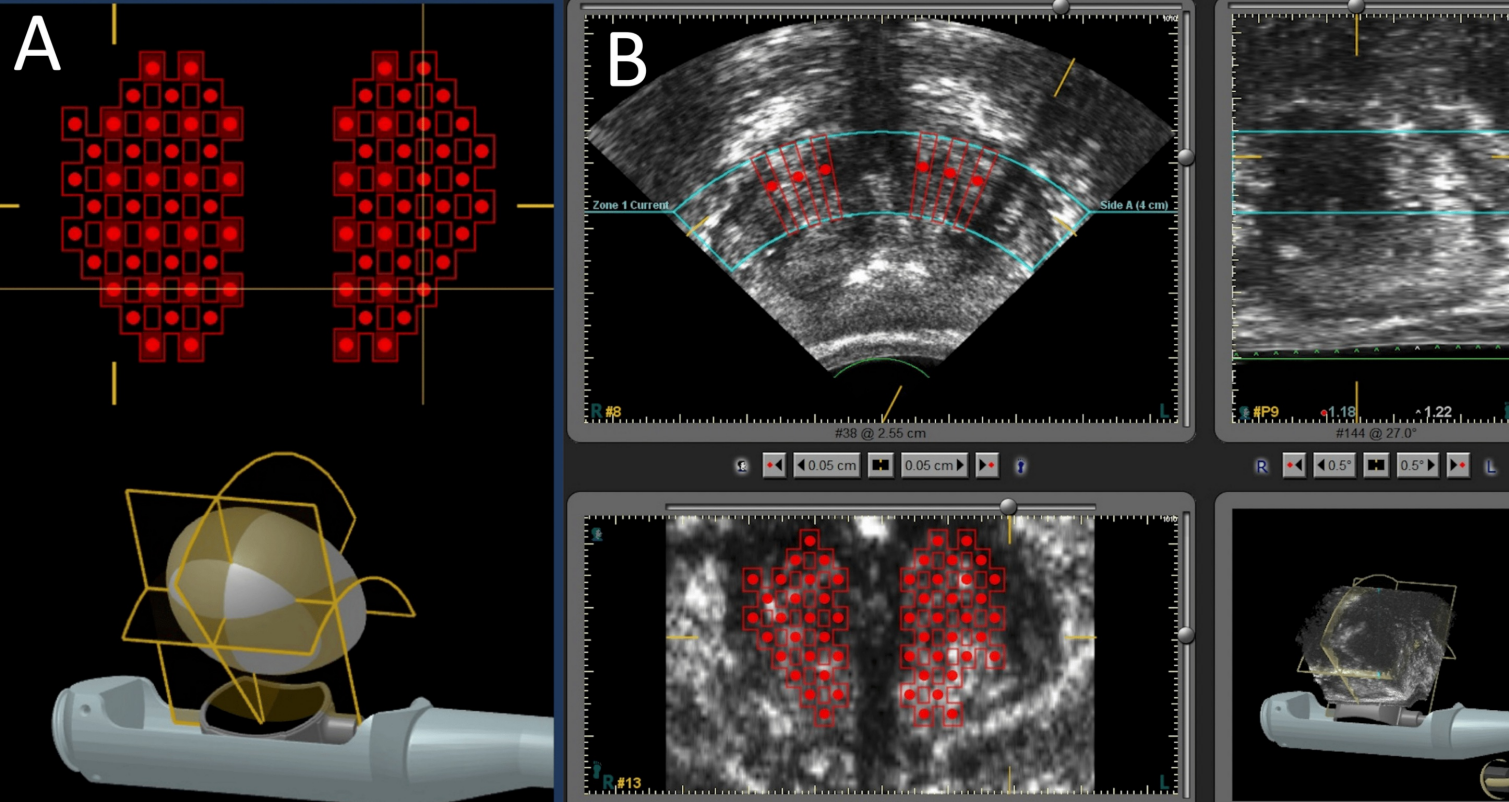
Representative images of the Histoblast technique treatment plan. A: Representative three-dimensional model of the transducer, the prostate, and the checkerboard treatment plan in the coronal plane. B: Screen from the high-intensity focused ultrasound (HIFU) device displaying a checkerboard treatment plan across axial, coronal, and sagittal planes.

The SB-500 transducer is introduced transrectally with the globe of the transducer already filled with ≈40 cc of degassed water, and the prostate is centered in both sagittal and axial scans using the device’s B-mode ultrasound imaging. Once the gland is located and centered, approximately 100 mL of degassed water is infused into the transducer to facilitate gland compression (Figure 1). Following prostate compression, the treatment plan is delimited within the transition zone with a plan following a checkerboard pattern (Figure 2A, 2B), with alternating treatment sites across anterior, mid, and/or posterior prostate zones, depending on gland volume. The number of treatment zones delivered in the procedure is assigned according to gland volume but follows the same pattern. Throughout the procedure, care is taken to spare the urethra, which is easily identifiable due to the catheter, as well as the bladder neck and apical portion of the enlargement.

Energy delivery uses the upper third of the approved power settings: up to 30 Watts for the 3 cm (Side B) transducer and up to 45 Watts for the 4 cm (Side A) transducer. During the procedure, the safety parameters used to modulate the energy remain the same as in conventional treatments (Tissue Change Monitoring, Reflectivity Index measurements, and hyperechoic Uchida Changes grade I or II, among others).

Upon completion, the transducer is removed, and patients are monitored in the recovery room until the anesthetic effects have worn off, after which they are discharged. Postoperatively, patients receive a progressive return to a regular diet, oral prophylactic antibiotics, and a urinary catheter for ≈4 days.

## Discussion

To the best of our knowledge, no other HIFU technique incorporating this specific combination of modifications has been previously described for the treatment of BPH.

Each of the three elements in this approach has individual support in the literature. Prostate compression has been reported to improve HIFU efficacy by reducing heat dissipation and enhancing tissue response, particularly in treatments using the SB-500 [13,14]. The energy settings applied in this protocol remain within the manufacturer’s approved safety margins and are typically adjusted intraoperatively based on tissue response indicators, such as changes in Tissue Change Monitoring or cavitation behavior [4]. The checkerboard pattern relies on both the well-characterized lateral heat diffusion of approximately 3 mm from the focal point [4] and, when combined with the other elements of the technique, enhances energy delivery efficiency, promoting greater mechanical ablation through increased acoustic cavitation [15].

Preliminary data previously presented [12] have shown that, with the current modifications, the procedure achieved boiling cavitation in approximately 70-75% of the total sonications for all patients, with the effect remaining confined to the planned treatment zone. However, these preliminary findings must be confirmed in prospective clinical studies.

The integration of these modifications (while preserving the urethra and bladder neck) may contribute to reduced edema, faster recovery of urinary function, and more efficient energy delivery without the complete set of shots. These theoretical advantages are grounded in known physical principles and are consistent with prior observations in related HIFU applications where whole-gland treatment could cause bladder outlet obstruction [9,10].

The main limitation of this article is that it provides only a technical report describing this novel cavitative HIFU technique; therefore, it does not present results on its clinical application or patient outcomes. Future prospective studies should evaluate these aspects and provide a comprehensive assessment in a clinical setting.

## Conclusions

This novel modification of the HIFU technique introduces procedural refinements aimed at optimizing energy delivery, reducing treatment time, and minimizing catheterization duration in BPH therapy. These features are grounded in well-established physical principles and findings previously described in the scientific literature. While the approach appears technically promising and addresses key limitations of conventional HIFU, further validation through a well-designed clinical trial is required to determine its clinical applicability.

## References

[1] Lokeshwar SD, Harper BT, Webb E (2019). Epidemiology and treatment modalities for the management of benign prostatic hyperplasia. Transl Androl Urol.

[2] Sanghvi NT, Foster RS, Bihrle R (1999). Noninvasive surgery of prostate tissue by high intensity focused ultrasound: an updated report. Eur J Ultrasound.

[3] Reddy D, Peters M, Shah TT (2022). Cancer control outcomes following focal therapy using high-intensity focused ultrasound in 1379 men with nonmetastatic prostate cancer: a multi-institute 15-year experience. Eur Urol.

[4] Barkin J (2011). High intensity focused ultrasound (HIFU). Can J Urol.

[5] Madersbacher S, Schatzl G, Djavan B, Stulnig T, Marberger M (2000). Long-term outcome of transrectal high- intensity focused ultrasound therapy for benign prostatic hyperplasia. Eur Urol.

[6] Uchida T, Muramoto M, Kyunou H, Iwamura M, Egawa S, Koshiba K (1998). Clinical outcome of high-intensity focused ultrasound for treating benign prostatic hyperplasia: preliminary report. Urology.

[7] Mulligan ED, Lynch TH, Mulvin D, Greene D, Smith JM, Fitzpatrick JM (1997). High-intensity focused ultrasound in the treatment of benign prostatic hyperplasia. Br J Urol.

[8] Schatzl G, Madersbacher S, Djavan B, Lang T, Marberger M (2000). Two-year results of transurethral resection of the prostate versus four 'less invasive' treatment options. Eur Urol.

[9] Byun SS, Jin N, Lee H (2022). High-intensity focused ultrasound ablation for prostate cancer: whole versus partial gland ablation. Clin Genitourin Cancer.

[10] Pan Y, Wang S, Liu L, Liu X (2022). Whole-gland high-intensity focused ultrasound ablation and transurethral resection of the prostate in the patients with prostate cancer: a systematic review and meta-analysis. Front Oncol.

[11] Valerio M, Ahmed HU, Emberton M (2014). The role of focal therapy in the management of localised prostate cancer: a systematic review. Eur Urol.

[12] Unno R (2023). Abstracts of the 40th World Congress of Endourology: WCE 2023. J Endourol.

[13] Shoji S, Hashimoto A, Nakamoto M (2015). Morphological analysis of the effects of intraoperative transrectal compression of the prostate during high-intensity focused ultrasound for localized prostate cancer. Int J Urol.

[14] Shoji S, Naruse J, Ohno S (2025). Focal therapy using high-intensity focused ultrasound with intraoperative prostate compression for patients with localized prostate cancer: a multi-center prospective study with 7 year experience. Prostate Cancer Prostatic Dis.

[15] Haar GT, Coussios C (2007). High intensity focused ultrasound: physical principles and devices. Int J Hyperthermia.

